# A multi-period robust portfolio optimization framework using yager’s entropy

**DOI:** 10.1371/journal.pone.0332725

**Published:** 2026-05-14

**Authors:** Arman Khosravi, Seyed Jafar Sadjadi

**Affiliations:** Department of Industrial Engineering, Iran University of Science and Technology, Tehran, Iran; University of Genoa: Universita degli Studi di Genova, ITALY

## Abstract

Modern portfolio theory, while foundational, struggles with extreme sensitivity to input parameters and a tendency to select highly concentrated portfolios, often leading to out-of-sample underperformance. This research addresses these critical gaps by introducing the Generalized Robust Mean-Variance-Entropy (GRMVE) framework, a novel Mixed-Integer Quadratic Programming (MIQP) model designed for adaptive, multi-period investment settings. The GRMVE framework integrates budgeted uncertainty to mitigate the risk of estimation errors in expected returns, while simultaneously incorporating Yager’s entropy to mathematically enforce structural diversification and prevent over-concentration. The framework is empirically validated using a dynamic, expanding-window re-optimization approach over a 72-month horizon on a pre-selected universe of 60 assets spanning all 11 sectors of the S&P 500. Out-of-sample results demonstrate that the GRMVE framework achieves highly competitive risk-adjusted returns compared to the famously resilient Naive (1/n) strategy and the state-of-the-art Mean-CVaR model. Crucially, it overcomes the severe portfolio concentration and prohibitive trading turnover associated with scenario-based tail-risk models (CVaR) and the extreme instability of non-robust mean-variance models. By successfully navigating severe market downturns and volatile recoveries with significantly lower drawdowns, the findings highlight the GRMVE framework as a highly practical, computationally tractable, and structurally resilient solution for institutional portfolio management.

## 1. Introduction

Modern portfolio theory, since its foundation by Markowitz [1], has provided the essential framework for portfolio selection through balancing risk and return [2]. While the classic mean-variance model offers theoretical elegance, its practical application is challenged by two persistent issues that can lead to suboptimal and unstable outcomes [3]. The first is a high sensitivity to input parameters; as demonstrated by Best and Grauer [4], small and statistically insignificant errors in expected return estimates can produce erratic and impractical portfolio allocations, a problem commonly known as “error maximization” [5]. The second is the tendency of optimization models to generate highly concentrated portfolios or “corner solutions,” which exposes investors to significant idiosyncratic risk and contradicts the fundamental principle of diversification. This concentration problem is so significant that a simple, equally-weighted “1/n” portfolio often outperforms sophisticated models in out-of-sample tests [6].

To address these limitations, two distinct areas of research have evolved. The first, robust optimization, confronts parameter uncertainty directly by constructing portfolios designed to perform well even under worst-case scenarios [7]. Among various approaches, the budgeted uncertainty framework introduced by Bertsimas and Sim [8] has emerged as a particularly powerful and practical method, offering an intuitive way to control conservatism without the excessive pessimism of earlier models [9]. The second area focuses on enhancing portfolio composition, often using entropy as a direct measure of diversification, an approach first applied to the portfolio selection problem by Philippatos and Wilson [10]. By incorporating an entropy-based objective, these models can explicitly prevent over-concentration in a few assets [11]. However, despite their individual advantages, these two fields of study have largely developed in parallel, leaving a critical gap in their integration.

This paper identifies and addresses two primary gaps in the portfolio optimization literature. The first is the discrepancy between the dynamic nature of financial markets and the static, single-period application of most robust models. An initially robust portfolio can become poorly positioned as market conditions change, a critical limitation in a fundamentally dynamic process [12]. There has been limited research integrating modern techniques like the budgeted uncertainty approach within an adaptive, multi-period framework. The second and more significant gap is the lack of a unified framework that formally and simultaneously tackles both parameter uncertainty and concentration risk. Without this integration, even robust models can remain vulnerable to unexpected events by selecting highly concentrated portfolios.

To bridge these gaps, this study develops and empirically validates a novel Generalized Robust Mean-Variance-Entropy (GRMVE) framework. The proposed model makes three core contributions: it operates within an adaptive, multi-period setting using a rolling-horizon strategy to dynamically adjust to new market information; it employs the budgeted uncertainty approach to manage the risk of inaccurate expected return estimates; and it formally integrates Yager’s entropy [13] as a tool to ensure portfolios remain well-diversified. Our numerical results demonstrate that this integrated approach provides a powerful and practical solution. Empirical tests show that the GRMVE framework successfully manages a volatile out-of-sample period, returning superior risk-adjusted performance compared to frameworks that neglect either robustness or diversification. Ultimately, our model achieves an attractive balance between mitigating downside risk and capturing market upside, delivering returns nearly identical to the famously resilient Naive (1/n) benchmark but with significantly higher stability.

The remainder of this paper is organized as follows. Section 2 reviews the foundational literature on multi-period portfolio optimization, robust optimization, and diversification measures, establishing the context for our research. Section 3 details the mathematical formulation of our proposed GRMVE framework and the benchmark models used for comparison. Section 4 presents the dataset, experimental design, and the numerical results of our empirical analysis. Section 5 discusses the interpretation and implications of our findings and acknowledges the study’s limitations, while Section 6 concludes the paper and offers directions for future research.

## 2. Literature review

In this section, we review the foundational literature that motivates our research. We begin by examining the development of multi-period portfolio optimization, contrasting the common strategies of static rebalancing and dynamic re-optimization. Next, we address the critical issue of input parameter uncertainty, which leads us to the field of robust optimization and its methods for building robust portfolios. Then we explore the challenge of portfolio concentration and review the use of entropy as a powerful tool for ensuring diversification. By systematically analyzing these key research areas, we will establish the context for our work and clearly identify the specific research gap that this paper aims to fill.

### 2.1. Multi-period portfolio optimization: from static rebalancing to dynamic re-optimization

The mean-variance (MV) model, introduced by Markowitz [1], serves as the foundation for single-period investment decisions. In practice, however, investors must frequently adjust their portfolios in response to changing market conditions. As Campbell and Viceira [12] highlight, asset allocation for long-term investors is fundamentally a dynamic process. Consequently, significant research has aimed to expand portfolio selection from a single-period to a multi-period context. The groundwork for this transition was established by researchers like Mossin [14], Merton [15], and Samuelson [16], who used dynamic programming to adapt the single-period model. Later work focused on real-world application, with Pogue [17] adding key factors such as transaction costs and taxes, while Elton and Gruber [18] investigated the relationship between multi-period and sequential single-period strategies. Since then, the literature has generally followed two main implementation methods: static rebalancing and dynamic re-optimization.

The most widely used strategy is rebalancing. This method involves setting an initial target for asset allocation and periodically returning the portfolio’s weights to that original target (e.g., [19,20]). The main benefit of rebalancing is its simplicity and its effectiveness in controlling portfolio drift. An important consideration in rebalancing is managing transaction costs, which include fixed fees, per-transaction commissions, and management fees based on portfolio value. Woodside-Oriakhi et al. [21] directly address these costs in their rebalancing models, which result in complex mixed-integer quadratic programs (MIQP). The continued relevance of this method is shown in recent work by Wong and Hsieh [22], who explore the impact of rebalancing frequency and transaction costs on the log-optimal portfolio. However, this simplicity has a major drawback. As Plaxco and Arnott [23] point out, a fixed rebalancing strategy is inherently static. Its inability to adapt is a critical weakness; by sticking to a historical asset mix, it ignores new market information, leading to missed opportunities and potentially poor long-term results.

A more advanced and flexible method is the re-optimization approach. With this technique, the entire portfolio optimization problem is solved again at the start of each new period using the latest available data and forecasts. This strategy is built on the ideas of multi-stage stochastic programming [24] and is a progression from earlier linear programming models for dynamic investing [25].

This period-by-period re-optimization is formally known in the broader optimization literature as the expanding-window approach. The expanding-window framework is a heuristic method specifically designed to address large, time-structured problems that are often too complex to be solved to global optimality over the entire planning period in one attempt [26]. The core idea is to decompose the long-term problem into a sequence of smaller, more manageable sub-problems that are solved iteratively. This approach repeatedly solves the model for a defined future timeframe, known as the forecast horizon, after which only the decisions for the immediate time step are implemented [27]. The horizon then “extends” forward, and the process is repeated with updated information. This structured and adaptive methodology is crucial for enabling the model to learn and adjust its portfolio strategy in response to new market information over time.

By re-optimizing, the model can actively change the portfolio’s structure to take advantage of new market information, allowing it to learn and adapt over time.

Despite being theoretically better, the re-optimization approach is used less often in robust optimization research than rebalancing. This is mainly due to the difficulties it presents. Creating a model for how uncertainty changes over many periods and solving a series of difficult optimization problems leads to major computational challenges, often called the “curse of dimensionality” [28]. To overcome this, some researchers have suggested using simpler, restricted policies like affine decision rules to make the problem more manageable (e.g., [29,30]). Although useful, these solutions are a trade-off, as they simplify the problem by sacrificing some of the adaptive power of full re-optimization.

Nevertheless, the field is increasingly adopting more powerful adaptive methods, thanks to progress in computing and data science. New developments in data-driven robust optimization are opening up possibilities for multi-stage problems [31]. For example, Ling et al. [32] created a multi-period robust portfolio model that includes dynamic risk management by controlling for downside risk. Additionally, the use of advanced forecasting tools like machine learning is at the forefront of this approach, where re-optimization at each step is guided by the latest return predictions [33].

Even with these improvements, the use of a period-by-period re-optimization strategy in robust frameworks, especially those using modern uncertainty models like the budgeted approach, has not been thoroughly investigated. While sophisticated dynamic models are being developed, a model that combines budgeted uncertainty with new diversification tools like Yager’s entropy has not yet been explored. This research gap presents an important opportunity to create a model that is both resistant to estimation errors and fully adaptable to changing financial markets, which is the main motivation for this paper.

### 2.2. Addressing uncertainty: the rise of robust optimization

A foundational challenge in modern portfolio theory is the classical Markowitz model’s extreme sensitivity to its input parameters. The model’s reliance on precise estimates for asset returns and covariances is a significant practical weakness, as these inputs are notoriously difficult to forecast. This issue was famously highlighted by Black and Litterman [34] and Best and Grauer [4], who demonstrated that minor, statistically insignificant changes in expected returns could trigger massive, unintuitive changes in the resulting “optimal” portfolio. This “error maximization” effect has been extensively quantified, with studies consistently concluding that uncertainty in expected returns is a far more significant source of error than uncertainty in the covariance matrix [5,35,36]. As Ben-Tal and Nemirovski [37] state, this acute sensitivity can render a theoretically optimal solution meaningless from a practical viewpoint, which has driven the development of techniques designed to be immune to such data uncertainty.

To confront this problem, two primary methodologies have emerged from the mathematical programming community: stochastic optimization and robust optimization [38]. Stochastic optimization requires knowledge of the exact probability distributions of the uncertain parameters [39], a demanding condition that is rarely met in practice and often leads to computationally intractable models. Robust optimization, in contrast, offers a more practical and deterministic alternative. The core principle of robust optimization is to define an uncertainty set, a bounded set containing all likely realizations of the uncertain data. The objective is then to find a solution that is protected against uncertainty by performing well even under the worst-case outcome within this set. This approach does not require full probability distributions and, as we will see, often leads to computationally tractable problems where the decision-maker can explicitly control the level of conservatism.

The application of the robust optimization framework in portfolio optimization has seen a clear evolution in how these uncertainty sets are constructed. The first major attempt by Soyster [9] introduced a box uncertainty set, where each parameter is assumed to lie within a simple interval. This approach considers the worst-case value for each parameter individually. However, it was quickly criticized for being overly conservative, as it implicitly assumes that all assets will simultaneously deliver their worst possible returns, a scenario that is highly improbable in practice [7,40].

To address this over-conservatism, a more refined approach using ellipsoidal uncertainty sets was pioneered by Ben-Tal and Nemirovski [7,37], El Ghaoui et al. [41], and Goldfarb and Iyengar [42]. An ellipsoidal set is statistically motivated and less conservative because it accounts for the covariance structure of the assets, making it impossible for all parameters to take their worst-case values at once. The resulting robust portfolio problem can be efficiently solved as a Second-Order Cone Program (SOCP), marking a significant advance in the field.

While powerful, the ellipsoidal approach still presents practical challenges related to its computational complexity and the difficulty of intuitively setting its single conservatism parameter. A highly influential framework that resolves these issues is the budgeted uncertainty approach, introduced by Bertsimas and Sim [8]. This method combines the simplicity of interval sets with an additional constraint, known as the “budget of uncertainty,” Γ. This budget parameter Γ (Γ∈[0, n] and not necessarily integer) directly controls how many asset returns are allowed to deviate from their nominal values at the same time. Bertsimas and Thiele [43], in a foundational comparison, argued that the primary weakness of the box uncertainty set is its extreme over-conservatism, a flaw that the budget of uncertainty approach directly fixes by limiting the number of parameters that can simultaneously take their worst-case values. Gregory et al. [44] highlighted the practical benefits of the budget of uncertainty in an international portfolio context, noting that its single, intuitive control parameter (Γ) was easier to set and adjust than the more abstract, statistically derived parameter required for an ellipsoidal set.

This elegant formulation has a crucial advantage, it preserves the original problem’s structure, meaning a robust linear program remains a linear program. This ensures computational tractability while giving the decision-maker an intuitive lever to adjust the trade-off between robustness and performance.

Sadjadi et al. [45] integrated the budget of uncertainty into a cardinality constrained portfolio problem, where their findings indicated that this specific robust approach could lead to better rates of return compared to portfolios formed using either box or ellipsoidal uncertainty sets. Bertsimas and Pachamanova [46] investigated a multi-period portfolio problem with transaction costs, where they found that the robust formulation not only remained computationally efficient but also selected portfolios with lower turnover, thereby reducing trading costs.

In summary, robust optimization provides a powerful and flexible framework for creating stable portfolios whose out-of-sample performance tends to be superior to that of classical mean-variance models [40]. Among the different robust optimization methodologies, the budgeted uncertainty approach stands out for its compelling combination of intuitive control, realistic conservatism, and computational efficiency. For these reasons, it provides a strong and well-justified foundation for the multi-period robust model developed in this research.

### 2.3. Addressing diversification: the role of entropy

A central goal in finance is to build portfolios that effectively manage risk through diversification. The core principle of diversification is that as the number of assets in a portfolio increases, the portfolio’s idiosyncratic (or unsystematic) risk can be reduced, ideally to zero. Therefore, a primary objective is to invest in as many mean-variance efficient assets as possible. However, a major issue with conventional portfolio selection models is their tendency to produce “corner solutions.” Corner solutions refer to portfolios that are highly concentrated in only a few assets. This low diversity makes the portfolio unstable and highly sensitive to small changes or errors in the input data [4]. While some have suggested that setting upper bounds on asset weights can reduce uncertainty, this often comes at the cost of sacrificing the performance of the diversified portfolio [47]. In a landmark study, DeMiguel et al. [6] showed that such optimized portfolios often fail to perform better out-of-sample than a simple, equally-weighted “1/n” strategy, further highlighting the practical challenges of concentration.

To directly address this problem, many researchers have proposed using entropy, a powerful concept that serves as a tool for measuring both risk and the degree of diversification [48]. Unlike the MV model, which uses the covariance matrix to manage risk, entropy provides a direct and straightforward measure of dispersion. It quantifies how widely the investment is spread across different assets. A portfolio with high entropy is, by definition, less concentrated and therefore more diversified. This approach is appealing because it does not depend on complex statistical assumptions about asset return distributions.

The most common entropy measure used in this field is Shannon’s Entropy [49]. It was first created to solve problems in communication theory and was later adapted for finance to measure the amount of information gained from market observations. Philippatos and Wilson [10] were the first to apply it to portfolio selection. For a portfolio containing n assets with corresponding weights $w_{i}$, Shannon’s entropy is calculated as:


$$H(w)=-\sum_{i=\text{1}}^{n}w_{i}\;\text{ln}\left(w_{i}\right)$$ (1)

According to Simonelli [50], using Shannon’s entropy can be a better way to construct a portfolio compared to using variance or similar measures of risk. It is frequently used as a main goal in optimization, sometimes even taking the place of variance [51], or as a rule to prevent a portfolio from becoming too concentrated. For example, Bera and Park [11] created a model with multiple goals that balanced mean, variance, and entropy. In a similar way, Usta and Kantar [52] added an entropy requirement to a model that also considered mean, variance, and skewness to create more diversified portfolios. This concept continues to be important in recent research, showing its lasting usefulness as a tool for managing how concentrated a portfolio is [53]. More recently, Mercurio et al. [54] conducted a comparative study showing that entropy-based portfolios can offer competitive risk-adjusted returns compared to traditional strategies, particularly in limiting downside risk. Despite its utility, the fixed mathematical form of Shannon’s entropy has led researchers to explore more flexible, generalized entropy measures. These alternatives, such as the entropies proposed by Rényi and Tsallis, introduce parameters that allow the measure of diversity to be adjusted, offering a more adaptable approach for financial markets.

Introduced in his seminal 1961 paper, Alfréd Rényi’s entropy measure offers a generalized way to quantify the diversity of a portfolio [55]. For a given portfolio with weights assigned to different assets, the Rényi entropy is calculated using a specific formula that incorporates a parameter, q. This parameter, which must be non-negative and not equal to one, plays a crucial role in adjusting the measure’s sensitivity to the shape of the portfolio’s weight distribution. By varying the value of q, an investor can place a different emphasis on various parts of the weight distribution, such as giving more or less weight to the tails of the distribution versus the center. This flexibility allows for a more nuanced approach to diversification, where the “amount of randomness” in a portfolio’s returns can be assessed to account for higher-order moments beyond simple variance [56].


$$H_{q}(w)=\frac{\text{1}}{\text{1}-q}\text{ln}\left(\sum_{i=\text{1}}^{n}\overset{q}{\underset{i}{w}}\right)$$ (2)

In the context of portfolio management, a higher Rényi entropy value is indicative of a more diversified portfolio [57]. Minimizing Rényi entropy can lead to portfolios that show better performance in terms of the trade-off between risk, return, and turnover compared to traditional minimum variance portfolios [56]. Lassance and Vrins [56] showed that the Rényi entropy of a portfolio’s return distribution not only offers a superior risk measure, leading to better out-of-sample performance, but also that its tunable parameter allows investors to control the trade-off between variance and kurtosis (tail risk).

In a 1988 paper, Constantino Tsallis proposed a non-extensive generalization of Shannon entropy, meaning it handles systems where the total entropy is not simply the sum of the entropies of its parts [58]. Its formula also includes a real parameter q, which determines the degree of this non-extensivity, and a positive constant k, which is often simplified to one [59]. This approach is considered useful for analyzing complex financial systems that may be far from equilibrium [60]. Similar to Rényi entropy, a higher Tsallis entropy value indicates greater portfolio diversification [54].


$$S_{q}(w)=\frac{k}{q-\text{1}}\left(\text{1}-\sum_{i=\text{1}}^{n}\overset{q}{\underset{i}{w}}\right)$$ (3)

The flexibility of Tsallis entropy is also evident in its special cases and makes it a versatile tool for constructing diversified investment strategies. Chortane and Naoui [61] showed that entropic models, specially Tsallis entropy, outperform the traditional mean-variance models in terms of diversification and, especially, extreme risk management.

Following this trend toward more flexible measures, this research focuses on Yager’s Entropy. While generalized entropies offer flexibility through parameter tuning, Yager’s entropy provides a distinct objective. In the context of portfolio selection, maximizing Yager’s entropy is equivalent to minimizing the geometric distance between the portfolio weight vector w and the uniform portfolio u = [1/n, ..., 1/n]. The general definition of Yager’s Entropy [13]:


$$Y(w)={-\left(\sum_{i=\text{1}}^{n}{\left|w_{i}-\frac{\text{1}}{n}\right|}^{p}\right)}^{\frac{\text{1}}{p}}$$ (4)

This formulation provides a clear and powerful objective, find a portfolio that is as close as possible to the perfectly diversified “1/n” portfolio, according to a specific distance metric defined by the parameter p≥1.


$$Y(w)=-\sum_{i=\text{1}}^{n}\left|w_{i}-\frac{\text{1}}{n}\right|$$ (5)

When the parameter p=1, the formula becomes a simpler linear type (Eq. 5), making it highly suitable for large-scale optimization problems [62]. Yu et al. [63] showed that the models with Yager’s entropy yield higher performance because they respond to the changes in market by reallocating assets more effectively than those with Shannon’s entropy or other measures.

The application of these advanced entropy concepts in sophisticated financial models is still in its early stages. Specifically, while Yager’s framework is recognized for its theoretical advantages, its effectiveness in multi-period models under uncertainty has not been properly studied. The present research seeks to address this critical gap. By integrating Yager’s flexible measure of diversification into a multi-period robust optimization framework, this study will explore its capacity to deliver more stable and effectively diversified investment strategies for long-term investors operating in uncertain market conditions.

### 2.4. Research gaps

Our review of the literature reveals two primary gaps that this research aims to address. The first critical gap relates to the time horizon of investment decisions. While single-period robust optimization is effective at creating portfolios that are protected against initial input errors, its static nature is a fundamental weakness in dynamic financial markets. A portfolio optimized for a single period can become poorly positioned as new market information becomes available, failing to adapt to new risks and opportunities. Although the benefits of robustness are well-established, there has been limited research integrating the flexible budget of uncertainty approach within an adaptive, multi-period framework that relies on period-by-period re-optimization.

A second, equally important gap exists between robustness and diversification. Robust optimization effectively addresses uncertainty in model parameters, but it does not inherently solve the problem of concentration risk. Optimization models, even robust ones, are still prone to selecting highly concentrated portfolios that are vulnerable to unexpected events not captured by the uncertainty set. The literature on robust optimization and the literature on diversification have largely developed in parallel. Consequently, there is a clear need for a framework that formally integrates these two objectives.

Therefore, this paper addresses these issues by developing and testing a model with the following key features:

- A multi-period setting that uses a re-optimization strategy to adapt to new market information over time.
- The use of robust optimization, specifically the budgeted uncertainty approach, to manage the risk of inaccurate return forecasts.
- The integration of Yager’s entropy as a formal mechanism to ensure portfolios remain well-diversified and avoid over-concentration in a few assets.

## 3. Methodology

This section details the strict mathematical framework developed to address the dual challenges of parameter uncertainty and portfolio concentration in a multi-period investment context. We begin by formally defining the multi-period portfolio optimization problem. Subsequently, we construct the core components of our proposed solution: a robust framework to protect the portfolio against adverse movements in expected returns, and a diversification measure derived from Yager’s entropy. Finally, we present the integrated proposed model and discuss its mathematical properties and solution approach.

### 3.1. Problem formulation

We first frame the general multi-period portfolio selection problem. The objective is to determine a sequence of portfolio compositions over a finite time horizon T that is optimal according to the Variance-Entropy objective function formulated using weighted sum approach, subject to a set of real-world constraints.

In this study, we manage the multi-period nature of the portfolio problem using a period-by-period “Re-Optimization” strategy. This approach is formally known in the optimization literature as an “Expanding-Window” framework. The framework decomposes the long-term investment problem into a series of sequential, short-term optimizations, allowing the model to adapt dynamically to new market information at each period.

Objective. The primary objective of an investor is to manage the trade-off between maximizing returns and minimizing risk over the investment horizon. Our model formulates this trade-off through a flexible, weighted objective function. It seeks to minimize a convex combination of portfolio variance (a traditional risk measure) and a tractable measure of portfolio concentration derived from Yager’s entropy, allowing for various levels of risk-diversification preferences.

Decision & Auxiliary Variables. The model solves for the following variables at each time period t∈{0, …, T} for each asset i∈{1, …, n}:

1. $x_{t,i}$: The proportion of the total portfolio value invested in asset i at the end of period t.
2. $y_{t,i}$: A binary variable, where $y_{t,i}=\text{1}$,if asset i is held in the portfolio during period t, and 0 otherwise. This is used to enforce cardinality constraints.
3. $s_{t,i}$: An auxiliary variable representing the absolute deviation of asset weight $x_{t,i}$ from the uniform weight 1/n.
4. $v_{t,i}$: The turnover in asset i during period t, representing the absolute value of the change in its weight from t−1 to t.

Constraints: The optimization is subject to a set of standard and practical constraints:

1. Budget Constraint: The portfolio must be fully invested at all times.
2. No Short-Selling: Selling assets that are not owned is prohibited.
3. Cardinality Constraints: The number of assets held in the portfolio is controlled to manage complexity and transaction costs.
4. Transaction Costs: Transaction costs are assumed to be a linear function of turnover, a common and practical model used by Pogue [17] and Woodside-Oriakhi et al. [21]. The turnover is defined as:

$$v_{t,i}=|x_{t,i}-x_{t-\text{1},i}|$$ (6)

*Assumptions:*

1. Re-Optimization occurs at discrete, equidistant time intervals.
2. The investment horizon T is finite.
3. The covariance matrix of asset returns is calculated at the beginning of each period t using historical data, and then remains constant for the optimization problem solved for that period.
4. Expected asset returns are uncertain and are modeled as belonging to a well-defined uncertainty set.

### 3.2. Constructing the integrated robust and diversification framework

To address the “error maximization” sensitivity of classical models [4], our methodology integrates two distinct but complementary frameworks drawn from the literature. First, we use the budgeted uncertainty approach of Bertsimas and Sim [8] to handle uncertainty in expected returns. Second, we embed a diversification measure based on Yager’s entropy [13] directly into the objective function to prevent the over-concentration issue of optimization models.

Robust Framework for Expected Returns. We adopt the budgeted uncertainty framework, which provides an intuitive and computationally efficient method for controlling the level of conservatism in the model. This approach avoids the excessive pessimism of early robust models that assume all parameters will simultaneously take their worst-case values [7].

The uncertainty for the expected return $\mu_{i}$ of each asset i is modeled as a symmetric interval $\left[\mu_{i}-d_{i},\;\mu_{i}+d_{i}\right]$, where $\mu_{i}$ is the nominal expected return and $d_{i}$ is the maximum deviation. The crucial innovation of the Bertsimas and Sim [8] framework is the introduction of a “Budget of Uncertainty,” Γ, which constrains the number of asset returns that can deviate from their nominal values at any given time. This creates a polyhedral uncertainty set. The worst-case return of the portfolio under this uncertainty model is protected by a robust counterpart constraint, which is formulated as a linear inequality and incorporated directly into our model (the first constraint of the proposed framework in Section 3.3).

The transformation from the classical model to its robust counterpart is detailed in 5 steps, available in the *appendix*.

The parameter Γ allows to control the trade-off between robustness and optimality. A value of Γ=0 recovers the classical model; while increasing Γ produces progressively more conservative portfolios that are protected against a larger number of simultaneous adverse shocks to returns.

Diversification Framework using Yager’s Entropy. To ensure that the portfolio is well-diversified, we leverage the concept of Yager’s entropy. As discussed in the literature review, unlike Shannon’s or Rényi’s entropy, Yager’s entropy provides a direct measure of the geometric distance between a portfolio vector w and the perfectly diversified uniform portfolio u=[1/n, ..., 1/n] [13]. Maximizing this entropy is equivalent to minimizing this distance.

For computational tractability, we employ the linear ($L_{\text{1}}$-norm) variant of this distance measure, which corresponds to Yager’s entropy for p=1 [62]. The objective is to minimize $\sum \left|x_{t,i}-\frac{\text{1}}{n}\right|$. To incorporate this into our optimization model, we use a standard linearization technique. We introduce a non-negative auxiliary variable $s_{t,i}$ for each asset and add the following two constraints:


$${𝐬}_{t,i}\geq x_{t,i}-\frac{\text{1}}{n}$$ (7)

$$s_{t,i}\geq -(x_{t,i}-\frac{\text{1}}{n})$$ (8)

By minimizing $\sum s_{t,i}$ in the objective function, we effectively minimize $\sum \left|x_{t,i}-\frac{\text{1}}{n}\right|$. This method, demonstrated to yield higher performance by Yu et al. [63], directly pushes the portfolio structure towards a more balanced allocation, thus promoting diversification.

### 3.3. The proposed multi-period robust portfolio optimization model

Integrating the elements from the preceding sections, we now present the complete formulation of our proposed Generalized Robust Mean-Variance-Entropy (GRMVE) framework. The GRMVE framework minimizes a weighted objective of portfolio variance and entropy-based diversification, subject to a robust return guarantee and other practical constraints.

*Model Formulation*

*Minimize:*


$$\lambda \sum_{i=\text{1}}^{n}\sum_{j=\text{1}}^{n}x_{t,i}x_{t,j}{Cov}_{t,i,j}+(\text{1}-\lambda )\sum_{i=\text{1}}^{n}s_{t,i}$$ (9)

*s.t*


$$\sum_{i=\text{1}}^{n}\mu_{t,i}x_{t,i}-\left(z\Gamma +\sum_{i=\text{1}}^{n}p_{t,i}\right)-\sum_{i=\text{1}}^{n}TCv_{t,i}\geq \mu_{\text{0}}$$ (10)

$$\sum_{i=\text{1}}^{n}x_{t,i}=\text{1}$$ (11)

$$L_{i}y_{t,i}\leq x_{t,i}\leq U_{i}y_{t,i}\;;\forall t$$ (12)

$$C_{Min}\;\leq \sum_{i=\text{1}}^{n}y_{t,i}\leq C_{max}$$ (13)

$$z+p_{t,i}\geq \;d_{i}x_{t,i}\;;\forall i$$ (14)

$$s_{t,i}\geq x_{t,i}-\frac{\text{1}}{n}$$ (15)

$$s_{t,i}\geq -(x_{t,i}-\frac{\text{1}}{n})$$ (16)

$$v_{t,i}\geq x_{t,i}-x_{t-\text{1},i}$$ (17)

$$v_{t,i}\geq -(x_{t,i}-x_{t-\text{1},i})$$ (18)

$$x_{t,i},\;z,\;p_{t,i},s_{t,i},v_{t,i}\geq \text{0}\;;\forall i$$ (19)

$$y_{t,i}\in \{\text{0},\text{1}\}\;;\forall i$$ (20)

The reference list of model components, parameters and notations are presented in Table 1.

**Table 1:** Reference List of Parameters and Variables.

*Parameters*	
λ	A parameter controlling the trade-off between minimizing risk (variance) and maximizing diversification (Yager’s entropy)
${Cov}_{i,j}$	The covariance between the returns of asset i and asset j
$\mu_{i},d_{i}$	The nominal expected return and maximum deviation for asset i
Γ	The budget of uncertainty for expected returns
$\mu_{\text{0}}$	The minimum required worst-case portfolio return
*TC*	The unit transaction cost
$L_{i}$, $U_{i}$	Lower and upper bounds for investment in asset i if held
$C_{min}$, $C_{max}$	Minimum and maximum number of assets to be held
*Decision Variables & Auxiliary Variables*	
$x_{t,i}$, $y_{t,i}$, $s_{t,i}$, $v_{t,i}$	As defined in Section 3.1
z, $p_{t,i}$	Auxiliary dual variables for the robust counterpart formulation

*Constraint Annotations:*

Objective Function: A convex combination of portfolio variance (risk) and the linear form (with p=1) of Yager’s entropy (diversification).
Robust Return Constraint: The robust counterpart of the return constraint. It guarantees that the portfolio return will be at least $\mu_{\text{0}}$ even after transaction costs, for the worst-case realization of returns within the uncertainty set defined by Γ.
(14) Robust Counterpart Auxiliary Constraint: A necessary constraint for the linearization of the robust formulation.
(15), (16) Yager’s Entropy Linearization: These constraints define $s_{t,i}$ as the absolute deviation of $x_{t,i}$ from 1/n.
(17), (18) Turnover Linearization: These constraints define $v_{t,i}$ as the absolute turnover in asset i.

### 3.4. Benchmark models

To evaluate the performance and practical advantages of the proposed GRMVE framework, we compare its numerical results against four distinct and widely recognized portfolio construction strategies. These benchmarks are chosen specifically to create a comprehensive comparison and to isolate the individual contributions of the robustness and entropy-based diversification components of our model. The strategies range from a simple heuristic to sophisticated optimization models that are special cases of our generalized framework. All these four benchmarks will inherit multi period setting to achieve unbiased results.

*The Naive (*$\frac{\text{1}}{n}$*) Portfolio:* The first benchmark is the naive $\frac{\text{1}}{n}$ diversification rule, which serves as a crucial, non-parametric baseline. This strategy invests an equal proportion, $\frac{\text{1}}{n}$, of capital into each of the n available assets at every period. Its primary advantage, as highlighted in the seminal work of DeMiguel et al. [6], is its complete immunity to estimation errors in the input parameters (μ and Cov), as it ignores them entirely. Despite its simplicity, the $\frac{\text{1}}{n}$ portfolio has been shown to be a surprisingly difficult benchmark to outperform, making it an essential test of any sophisticated optimization model’s practical utility.

The $\frac{\text{1}}{n}$ portfolio is not an optimization model, instead it’s a heuristic strategy which is defined by the simple rule for each period t:


$$x_{t,i}=\;\frac{\text{1}}{n};\text{for all }i=\;\text{1},\;...,\;n$$ (21)

This strategy satisfies the two classic constraints of the Mean-Variance model, the budget and no-short-selling constraints. However, it does not adhere to any of the other practical constraints handled by our proposed framework, such as cardinality, transaction costs, or floor and ceiling limits.

The Mean-Variance-Entropy (MVE) Model. The second benchmark is a sophisticated optimization model designed to isolate the contribution of our robustness framework. The Mean-Variance-Entropy (MVE) model is identical to our proposed GRMVE framework in every aspect including the use of the Yager’s entropy diversification term, except that it omits the robust optimization components. It relies on the nominal point estimates for expected returns, following the classical approach of Markowitz [1] but extended with modern practical constraints and an entropy-based objective. By comparing the GRMVE framework to this non-robust MVE counterpart, we can directly quantify the value added by protecting the portfolio against parameter uncertainty. The MVE model is formulated for each period t as follows:

*Objective Function*

*Minimize:*


$$\lambda \sum_{i=\text{1}}^{n}\sum_{j=\text{1}}^{n}x_{t,i}x_{t,j}{Cov}_{t,i,j}+(\text{1}-\lambda )\sum_{i=\text{1}}^{n}s_{t,i}$$ (22)

*Subject to:*

1. Standard Return Constraint:

*Minimize:*


$$\sum_{i=\text{1}}^{n}\mu_{t,i}x_{t,i}-\sum_{i=\text{1}}^{n}TCv_{t,i}\geq \mu_{\text{0}}$$ (23)

1. All Other Constraints: The MVE model is subject to the same budget, cardinality, floor/ceiling, entropy auxiliary, and turnover linearization constraints as the GRMVE framework.

The Robust Mean-Variance (RMV) Model. The third benchmark, the Robust Mean-Variance (RMV) model, is designed to isolate the contribution of our Yager’s entropy diversification component. This model fully incorporates the robust optimization framework to protect against uncertainty in expected returns but omits the entropy term from objective function. It is a direct implementation of a modern robust optimization strategy with practical constraints. By comparing the GRMVE framework to this RMV benchmark, we can precisely measure the impact of adding the explicit diversification measure on portfolio characteristics such as concentration, turnover, and risk-adjusted performance.

The RMV model is formulated for each period t as a special case of the GRMVE framework where λ=1:

*Objective Function:*

*Minimize:*


$$\sum_{i=\text{1}}^{n}\sum_{j=\text{1}}^{n}x_{t,i}x_{t,j}{Cov}_{t,i,j}$$ (24)

*Subject to:*

1. Robust Return Constraint:

$$\sum_{i=\text{1}}^{n}\mu_{t,i}x_{t,i}-\left(z\Gamma +\sum_{i=\text{1}}^{n}p_{t,i}\right)-\sum_{i=\text{1}}^{n}TCv_{t,i}\geq \mu_{\text{0}}$$ (25)

1. All Other Constraints: The RMV model is subject to the same robust auxiliary, budget, cardinality, floor/ceiling, and turnover linearization constraints as the GRMVE framework. The s variables and entropy-related auxiliary constraints are omitted.

The Mean-CVaR (Conditional Value-at-Risk) Model. To better contextualize the performance of the proposed GRMVE framework against state-of-the-art (SOTA) portfolio optimization techniques, we introduce the Mean-CVaR model as our fourth benchmark. While the classic mean-variance framework penalizes both upside and downside deviations equally, CVaR focuses exclusively on tail risk, measuring the expected loss that exceeds the Value-at-Risk (VaR) threshold. By incorporating this SOTA scenario-based risk management model, we can directly compare our budgeted uncertainty and entropy-based approach against a highly sophisticated, widely adopted modern benchmark.

Following the seminal linear formulation by Rockafellar and Uryasev [64], the Mean-CVaR model minimizes the conditional expected loss at a specified confidence level α (e.g., α=0.95), subject to a target return and practical constraints. To formulate this for each period t, we rely on S historical return scenarios, where $r_{t,i,s}$ represents the observed return of asset *i* in scenario *s* at the time of optimizing for period *t*.

*Objective Function:*

*Minimize:*


$$\eta_{t}+\frac{\text{1}}{(\text{1}-\alpha )S}\sum_{s=\text{1}}^{S}u_{t,s}$$ (26)

*Subject to:*

1. Expected Return Constraint:

$$\sum_{i=\text{1}}^{n}\mu_{t,i}x_{t,i}-\sum_{i=\text{1}}^{n}TCv_{t,i}\geq \mu_{\text{0}}$$ (27)

1. CVaR Shortfall Constraints

$${𝐮}_{t,s}\geq -\sum_{i=\text{1}}^{n}r_{t,i,s}x_{t,i}-\eta_{t}\;;\forall s$$ (28)

$$u_{t,s}\geq \text{0}\;;\forall s$$ (29)

1. All Other Constraints: The Mean-CVaR model is subject to the same budget, cardinality, floor/ceiling, and turnover linearization constraints as the GRMVE framework (Equations 11–13 and 17–20). The robust auxiliary and entropy-related constraints are omitted.

### 3.5. Solution approach

The proposed optimization model, as formulated in Section 3.3, is a Mixed-Integer Quadratic Program (MIQP) problem. The quadratic nature arises from the variance term (product of two decision variables $x_{i}x_{j}$) in the objective function, and the integer nature arises from the binary variable $y_{t,i}$ used to enforce cardinality and buy-in constraints.

Solution Algorithm. The model does not require a custom-designed algorithm and can be solved directly using modern solvers.

Implementation. The model will be implemented in Python. The Gurobi optimization solver, accessed through its Python API, will be used to solve the MIQP problem instances in the numerical experiments. This combination provides a powerful and flexible environment for implementing and testing the proposed model.

## 4. Numerical results

In this section, we present the numerical results that demonstrate the effectiveness and practical advantages of our proposed framework. The analysis is structured as follows: first, we describe the dataset and the motivation behind the performance metrics selected to evaluate the models on risk, return, and practical considerations like turnover. Second, we report and analyze the detailed results of the optimization, comparing our GRMVE framework against the naive, MVE, RMV and Mean-CVaR benchmark models to highlight the contributions of robustness and diversification. Finally, the performance of the model is examined under various parameter settings to analyze its sensitivity and to understand the trade-offs inherent in its design.

### 4.1. Dataset

To empirically evaluate the practical effectiveness and generalizability of the proposed framework across diverse market conditions, this study utilizes a comprehensive dataset of 60 highly liquid large-cap stocks. These assets were systematically selected to represent all 11 Global Industry Classification Standard (GICS) sectors of the S&P 500. This multi-sector approach ensures that the optimization models are tested against a diverse set of risk-return profiles and dynamic correlation structures, mitigating the risk of sector bias. The dataset comprises 72 months of historical monthly returns for these 60 assets, sourced from investing.com.

To simulate a realistic, institutional-grade quantitative investment process, we introduce a dynamic pre-selection stage prior to the rigorous mathematical optimization. In practice, feeding a massive asset universe directly into a Mixed-Integer Quadratic Program (MIQP) can lead to noise over-fitting and computational complexity. Therefore, at the beginning of each re-optimization period, we evaluate the 60-asset universe using the Calmar Ratio. The Calmar Ratio is a risk-adjusted performance metric that measures the historical annualized return of an asset relative to its maximum downside risk. For each asset i, the Calmar Ratio (${CR}_{i}$) is calculated as:


$${Calmar\;Ratio}_{i}=\frac{R_{Ann,i}}{\left|{MDD}_{i}\right|}$$ (30)

Where $R_{Ann,i}$ is the annualized compound return of asset i, and ${MDD}_{i}$ is the Maximum Drawdown of asset i over the historical period, defined as the maximum observed percentage drop from a historical peak to a subsequent trough.

This metric effectively screens for assets that demonstrate strong historical momentum while actively penalizing those with extreme downside volatility. Only the top 20 assets with the highest Calmar Ratios are selected to form the feasible asset universe fed into the proposed GRMVE framework and the benchmark models for that specific period.

Our analysis spans the 72-month timeframe using a dynamic, expanding window Re-Optimization approach. Rather than using a fixed-length rolling horizon that discards older data, this recursive method allows the models to incorporate all available historical information up to the moment of decision.

Specifically, the initial optimization (Period 0) utilizes the first 54 months of historical data to estimate the model inputs (e.g., expected returns, covariance matrix, and the Calmar pre-selection rankings). The optimal portfolio constructed from the selected 20 assets is then held and evaluated over the subsequent 6-month out-of-sample period. At the end of this period, the in-sample estimation window expands by 6 months. Thus, the re-optimization for Period 1 utilizes 60 months of historical data, and the final re-optimization for Period 2 utilizes 66 months of data.

**Fig 1:**
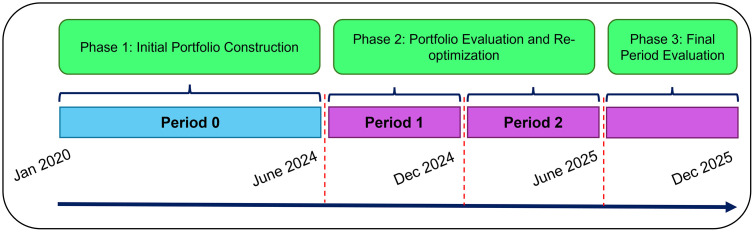
Timeline of the Portfolio Optimization and Evaluation Process.

In each period, the portfolio is evaluated strictly on the unseen 6 months immediately following the estimation window. This process results in three consecutive out-of-sample testing periods, yielding 18 months of continuous out-of-sample performance data. This expanding window framework allows us to rigorously assess how each strategy dynamically adapts its sectoral exposure and asset allocations as its historical knowledge base grows and market conditions evolve. A visual representation of this expanding-window timeline across the three evaluation periods is provided in Fig 1.

**Fig 2:**
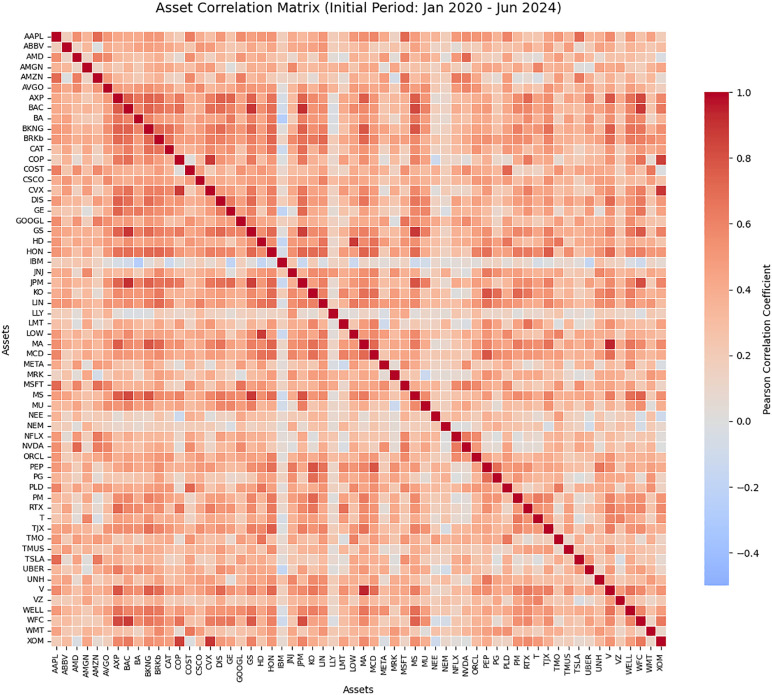
Correlation heatmap of the 60-asset universe spanning 11 S&P 500 sectors.

To understand the broad investment environment and the underlying diversification potential available to our models, we first analyze the correlation structure of the complete 60-asset universe. Because this universe spans all 11 GICS sectors of the S&P 500, it contains a vast and complex web of relationships. Feeding a covariance matrix of this size directly into a standard quadratic optimizer often leads to computational inefficiency and severe “error maximization.” This reality directly motivates our two-stage methodology, first, using the dynamic Calmar Ratio pre-selection to filter this universe down to the 20 most efficient assets per period, and second, applying our robust optimization framework. To illustrate the scale of this broad investment environment, Fig 2 presents the complete pairwise correlation heatmap for the 60-asset universe.

Observing this comprehensive heatmap (see Fig 2) highlights the critical challenge of modern portfolio construction. The matrix reveals distinct, dense blocks of high positive correlation (dark red/warm cells), which typically represent intra-sector relationships where traditional diversification benefits are minimal. Conversely, the matrix also displays regions of neutral or negative correlations (lighter/cool cells) across different asset classes, presenting distinct, high-value optimization opportunities.

Navigating this dense, noisy, and highly interconnected correlation structure using classical point-estimate models inherently leads to unstable portfolios. This complex market reality serves as the primary motivation for the proposed GRMVE framework, which utilizes Yager’s entropy to mathematically force broad diversification across these clusters, while employing the budgeted uncertainty approach to ensure that the model doesn’t over-rely on these highly sensitive pairwise historical estimates.

### 4.2. Performance metrics

To evaluate the performance of our proposed model and the benchmarks, we employ a set of four distinct metrics. These metrics provide a full view of each strategy, covering not only risk-adjusted returns but also portfolio concentration and practical trading costs.

*The Sharpe Ratio* [65] is the most widely recognized metric for measuring risk-adjusted return. It quantifies the amount of excess return an investor receives for each unit of total risk taken, where risk is measured by the standard deviation of returns (volatility). A higher Sharpe Ratio indicates better performance, as it implies a more efficient portfolio that generates higher returns for the risk it entails:


$$Sharpe\;Ratio=\;\frac{R_{p}-R_{f}}{\sigma_{p}}$$ (31)

Where $R_{p}$ is the expected return of portfolio, $R_{f}$ is the risk-free rate and $\sigma_{p}$ is the standard deviation of the portfolio’s excess returns.

*The Sortino Ratio* [66] is a modification of the Sharpe Ratio that separates downside volatility and upside volatility. Investors are generally not concerned with unexpected high returns. The Sortino Ratio, therefore, only penalizes a portfolio for returns less than a minimum acceptable return, typically the risk-free rate:


$$Sortino\;Ratio=\;\frac{R_{p}-R_{f}}{\sigma_{d}}$$ (32)

Where $\sigma_{d}$ is the downside deviation, or the standard deviation of only the negative excess returns.

*The Herfindahl-Hirschman Index (HHI)* is a standard measure of market concentration that we adapt to measure portfolio concentration. It helps to quantify how diversified a portfolio is. The HHI index is calculated by sum of the squared weight of each asset in the portfolio. HHI index ranges from [$\frac{\text{1}}{n},\;\text{1}$], where a lower value indicates a more diversified portfolio, and a higher value indicates a more concentrated one. An HHI of 1 represents a portfolio with 100% of its capital in a single asset. This metric is essential for assessing the effectiveness of the entropy-based component of our model:


$$HHI=\sum_{i=\text{1}}^{n}\overset{\text{2}}{\underset{i}{x}}$$ (33)

*Turnover* (as explained in 3.1) is a measure of the trading activity required to manage a portfolio between periods of the investment horizon. High turnover is undesirable as it leads to higher transaction costs which can significantly reduce the net return of a strategy.

### 4.3. Results & performance comparison

The core of our computational analysis is a comparative evaluation designed to quantify the practical advantages of the proposed GRMVE framework relative to established benchmarks. The objective of this analysis is to empirically validate the benefits of integrating robust optimization framework and Yager’s entropy diversification by comparing the models’ out-of-sample results across the suite of metrics defined previously.

For this core comparison, all optimization strategies were implemented under a consistent set of realistic constraints and parameters. The objective function was balanced with λ=0.5. For the robust models, we defined the uncertainty set using a standard and intuitive approach. The potential deviation for each asset’s return, $d_{i}$, was set to one standard deviation (σ) of its historical returns, directly linking the size of the uncertainty to the asset’s observed volatility. Subsequently, the budget of uncertainty Γ was set to 5. The selection of the budget of uncertainty (Γ) is a critical parameter that directly controls the model’s level of conservatism. A key motivation for the budgeted uncertainty framework was to overcome the excessive pessimism of earlier robust methods. These earlier approaches, such as Soyster’s, are equivalent to setting Γ equal to the total number of assets, which implicitly assumes the highly improbable scenario where all assets simultaneously realize their worst-case returns. For our core analysis, we have selected Γ=5. This choice represents a moderately conservative stance; it protects the portfolio against a scenario where up to five assets, a significant portion of the portfolio, deviate adversely without being so restrictive that it sacrifices all potential for performance. This specific setting allows for a meaningful evaluation of the model’s practical benefits, with the full impact of Γ explored later in the sensitivity analysis.

#### 4.3.1. Portfolio composition and structural stability.

**Fig 3:**
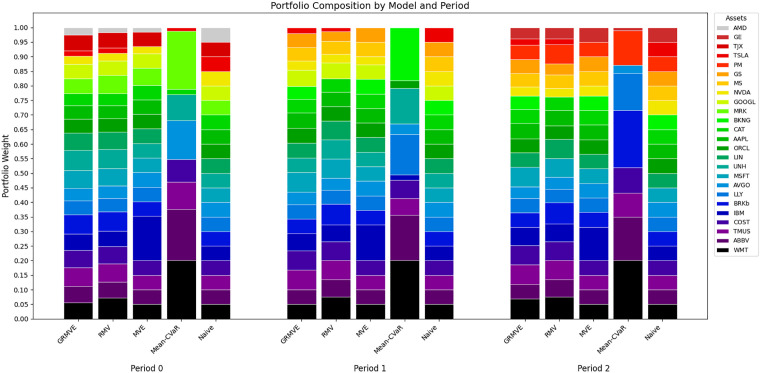
Dynamic Portfolio Composition by Model across the three Re-Optimization Periods.

Since our methodology utilizes a dynamic pre-selection stage, the feasible asset universe updates at each re-optimization period. To analyze the structural behavior and allocation logic of each strategy without relying on static ticker-level tables, we visualize the dynamic weight distributions via 100% stacked bar charts. Figure 3 presents the portfolio compositions for all five models across the three out-of-sample periods. Each distinct color band represents the weight allocated to a specific asset.

The baseline Naive (1/n) portfolio is represented by perfectly equal, static 5% allocations across all 20 assets. Among the models, the Mean-CVaR model exhibits extreme concentration. As visually evident across all three periods, the Mean-CVaR model aggressively allocates capital into a narrow subset of assets, frequently hitting the maximum upper bound constraint while completely excluding numerous others. This visualizes the classic “corner solutions” inherent to scenario-based tail-risk models, explaining its uniquely high HHI and trading turnover as it drastically reshuffles these large blocks between periods.

Conversely, the variance-based models systematically suppress this extreme concentration. The non-robust MVE and purely robust RMV models construct more balanced portfolios, though they still exhibit moderate concentration in favored assets.

Crucially, the proposed GRMVE framework achieves the most structurally balanced allocation among all the optimization models. Driven by its Yager’s entropy objective, the GRMVE model actively resists over-concentration, producing a visual profile that closely mirrors the perfectly diversified Naive strategy. It maintains exposure to nearly the entire 20-asset universe (represented by the high number of narrow, evenly distributed color bands) while smoothly and dynamically adjusting those weights across periods to optimize risk-adjusted returns. This visual evidence perfectly confirms that the GRMVE framework successfully integrates robust optimization with highly practical, real-world diversification.

#### 4.3.2. Out-of-sample performance.

Table 2 presents the detailed out-of-sample monthly returns for the 18-month evaluation horizon. By expanding the testing window, the models were subjected to a diverse set of market regimes, including a period of steady growth (late 2024), a severe market downturn (March 2025), and a volatile, erratic recovery phase (mid-to-late 2025). Analyzing the models’ behavior across these distinct phases provides critical insights into their structural advantages and limitations.

**Table 2:** Out-of-Sample Returns of Models.

Date	GRMVE	RMV	MVE	Mean-CVaR	Naive
2024-07-01	0.0103	0.0100	0.0057	0.0150	0.0079
2024-08-01	0.0472	0.0488	0.0452	0.0634	0.0411
2024-09-01	0.0272	0.0259	0.0290	0.0166	0.0379
2024-10-01	−0.0104	−0.0103	0.0024	−0.0110	−0.0130
2024-11-01	0.0469	0.0484	0.0343	0.0324	0.0530
2024-12-01	−0.0246	−0.0237	−0.0226	0.0147	−0.0132
2025-01-01	0.0323	0.0374	0.0317	0.0368	0.0280
2025-02-01	0.0114	0.0128	0.0208	0.0478	0.0003
2025-03-01	−0.0674	−0.0598	−0.0686	−0.0566	−0.0714
2025-04-01	0.0049	−0.0025	0.0032	0.0210	0.0087
2025-05-01	0.0414	0.0282	0.0353	−0.0303	0.0519
2025-06-01	0.0539	0.0475	0.0574	0.0284	0.0558
2025-07-01	0.0124	0.0115	0.0130	−0.0227	0.0156
2025-08-01	0.0173	0.0197	0.0149	0.0343	0.0191
2025-09-01	0.0592	0.0596	0.0623	0.0275	0.0714
2025-10-01	−0.0125	−0.0157	−0.0164	−0.0264	−0.0042
2025-11-01	0.0088	0.0140	0.0038	0.0840	0.0052
2025-12-01	0.0044	0.0003	0.0088	−0.0108	0.0071

The Downturn Stress Test (March 2025): The most significant market shock occurred in March 2025, serving as a critical stress test for the models’ risk management capabilities. The heuristic Naive (1/n) strategy, completely exposed to systemic market beta, suffered the most severe drawdown, losing −7.14% in a single month. The non-robust MVE model also fared poorly (−6.86%), failing to protect capital due to its reliance on point estimates. In contrast, the tail-risk focused Mean-CVaR model successfully insulated the portfolio, recording the smallest loss (−5.66%), closely followed by the purely robust RMV model (−5.98%). The proposed GRMVE framework effectively balanced its diversification mandate with its robust constraints, mitigating the shock (−6.74%) far better than the Naive benchmark.

The Cost of Extreme Conservatism: While the Mean-CVaR model excelled during the March crash, its performance during the subsequent market recovery highlights a severe practical limitation: extreme sensitivity and over-concentration in historically “safe” assets. In May, July, and December of 2025, the broader market (represented by the positive returns of the Naive strategy) and all variance-based models experienced positive growth. However, the Mean-CVaR model generated anomalous negative returns (−3.02%, −2.27%, and −1.07%, respectively). This indicates that the Mean-CVaR model became trapped in overly defensive, concentrated corner solutions that completely failed to capture the broader market’s upside, a classic regime-shift vulnerability often observed in scenario-based tail-risk models.

The GRMVE Framework’s Balanced Trajectory: The proposed GRMVE framework demonstrated exceptional resilience and adaptability throughout the 18-month period. It successfully avoided the unhedged drawdowns of the Naive strategy during the March crash, while completely avoiding the erratic, false-negative returns that plagued the Mean-CVaR model during the recovery. By systematically integrating budgeted uncertainty (to protect against the crash) and Yager’s entropy (to force broad market participation and prevent CVaR-like concentration), the GRMVE model captured consistent upside in months like May (+4.14%), June (+5.39%), and September (+5.92%).

Ultimately, the month-by-month analysis proves that while the Naive strategy offers high baseline returns at the cost of extreme risk, and the Mean-CVaR model offers extreme protection at the cost of market participation, the GRMVE framework delivers a highly consistent, computationally stable, and balanced investment trajectory.

**Fig 4:**
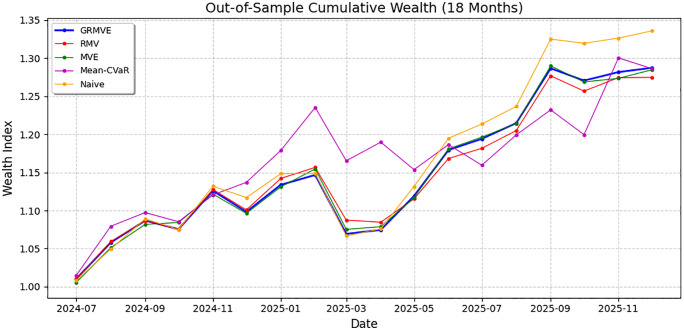
Out-of-Sample Cumulative Wealth of the models over the 18-month period.

Fig 4 illustrates the net cumulative growth of a hypothetical $1 investment in each strategy over the 18-month out-of-sample horizon. This visualization, which accounts for transaction costs incurred during each expanding-window re-optimization, provides critical insight into how the models navigate compounding growth, market shocks, and recovery phases.

The terminal wealth and the path taken to achieve it reveal a clear hierarchy of structural stability among the models. As expected, the heuristic Naive (1/n) portfolio achieved the highest terminal wealth (ending near 1.33). However, this superiority is primarily driven by its unhedged exposure to market beta during bullish phases, completely ignoring real-world portfolio constraints, cardinality limits, and targeted risk levels.

Among the sophisticated mathematical optimization models, the Mean-CVaR strategy exhibited the most volatile and erratic wealth trajectory. While it initially led the pack in early 2025, its intense focus on tail-risk minimization caused severe volatilities and underperformance. The Mean-CVaR model frequently concentrated capital into overly defensive assets, causing it to suffer sharp drawdowns precisely when the rest of the market (and the other models) were recovering, most notably in May, July, and December 2025. This visualizes a classic flaw in scenario-based models, over-fitting to historical tail-risk scenarios can extremely weaken out-of-sample compounding growth.

In contrast, the variance-based models (MVE, RMV, and GRMVE) followed a much tighter, more consistent trajectory. The non-robust MVE and the purely robust RMV models tracked closely together, but RMV ultimately suffered from “volatility drag,” finishing with the lowest terminal wealth among the group. Its strict focus on worst-case parameter deviations, without an explicit diversification mandate, proved too conservative to fully capitalize on the 18-month market expansion.

Ultimately, the proposed GRMVE framework emerged as the top-performing constrained optimization model. The blue GRMVE line demonstrates the balance achieved by our integrated approach. By relying on budgeted uncertainty, it avoided the extreme erratic swings of the Mean-CVaR model. By enforcing Yager’s entropy, it maintained broad enough market participation to outpace both the purely robust (RMV) and purely nominal (MVE) models by the end of the 18-month period. It successfully captured the majority of the market’s upside, delivering the highest terminal wealth among the optimization-based models via a significantly more stable, reliable, and practically implementable path.

**Fig 5:**
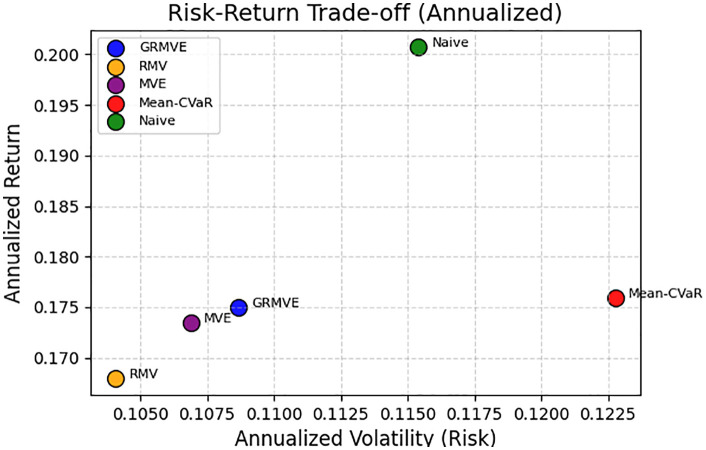
Risk-Return Performance.

Fig 5 visualizes the annualized risk-return efficiency of each strategy over the 18-month out-of-sample period, where the theoretical optimal position lies in the top-left quadrant (maximizing return while minimizing volatility). The plot reveals a stark contrast in structural behavior between the models. The heuristic Naive portfolio achieved the highest annualized return but incurred substantial volatility, reflecting its unhedged exposure to broader market beta. Conversely, the state-of-the-art Mean-CVaR model proved to be highly inefficient in a standard mean-variance context. While CVaR is explicitly designed to minimize extreme tail losses, its frequent and intense re-allocation into concentrated, defensive assets ultimately subjected the portfolio to the highest overall annualized volatility of any strategy, yielding only moderate returns.

Among the constrained optimization models, the proposed GRMVE framework demonstrates a clearly superior balance. The purely robust RMV model successfully anchored the low-risk end of the spectrum but sacrificed significant upside, resulting in the lowest annualized return. The non-robust MVE model offered slight improvements but remained suboptimal. The GRMVE framework, however, effectively shifted its performance upward into a highly favorable spot. By formally integrating budgeted uncertainty with Yager’s entropy, GRMVE achieved annualized returns nearly identical to the highly volatile Mean-CVaR model, yet it accomplished this while maintaining a strictly controlled, conservative risk profile nearly identical to the RMV and MVE models. This visually confirms that the GRMVE framework delivers the most efficient, risk-adjusted compromise among the tested mathematical models.

The preceding analysis examined the portfolio compositions and the overall risk-return performance of each strategy. Now, in this final stage of our analysis, we use the metrics defined in Section 4.2 to conduct a quantitative evaluation. This allows us to make a comprehensive and conclusive comparison between the proposed GRMVE framework and the benchmarks.

The visual trajectories of the 18-month out-of-sample period are formally quantified in Table 3, which consolidates the strategies’ total and annualized returns, risk-adjusted performance (Sharpe and Sortino Ratios), extreme risk (Max Drawdown), portfolio concentration (Average HHI), and practical trading costs (Average Turnover).

**Table 3:** Out-of-Sample Performance Metrics.

	Total Return	Ann. Return	Sharpe Ratio	Sortino Ratio	Maximum Drawdown	Avg. HHI	Avg. Turnover
GRMVE	0.2871	0.1750	1.6105	2.9123	−0.0674	0.0529	0.3048
RMV	0.2746	0.1680	1.6139	3.0666	−0.0622	0.0561	0.2669
MVE	0.2845	0.1735	1.6231	2.8683	−0.0686	0.0600	0.2611
Mean-CVaR	0.2861	0.1760	1.4333	2.8867	−0.0660	0.1466	0.7727
Naive	0.3356	0.2008	1.7397	3.3276	−0.0714	0.0500	0.2500

A comprehensive analysis of these metrics reveals the profound practical advantages of the GRMVE framework, particularly when contrasted against the state-of-the-art Mean-CVaR model and the non-robust benchmarks.

Risk-Adjusted Performance and Downside Protection: The fundamental goal of robust optimization is to protect against severe market shocks without sacrificing long-term compounding growth. The data clearly demonstrates the inherent trade-offs in this pursuit. The purely heuristic Naive (1/n) portfolio generated the highest Total Return (33.56%) and Sharpe Ratio (1.7397). However, this performance was achieved by assuming unhedged market beta, resulting in the most severe Maximum Drawdown (−7.14%) among all strategies. Conversely, the purely robust RMV model proved to be the absolute safest model, recording the shallowest drawdown (−6.22%) and a highly competitive Sortino Ratio (3.0666), but this extreme conservatism dragged its Total Return down to the lowest of the group (27.46%).

The proposed GRMVE framework established a highly effective middle ground. It significantly improved upon the total return of the purely robust RMV strategy (28.71% vs. 27.46%) while managing tail-risk far more effectively than the non-robust MVE model (mitigating the maximum drawdown to −6.74% compared to MVE’s −6.86%). Its Sharpe Ratio (1.6105) and Sortino Ratio (2.9123) confirm that the model delivers highly efficient, risk-adjusted growth that tightly competes with both MVE and RMV, but with a structurally superior portfolio.

The metrics expose a critical practical limitation of the Mean-CVaR model. While CVaR is explicitly designed to minimize tail risk, and successfully achieved a strong drawdown metric of −6.60%, it did so at a severe cost to overall efficiency. The Mean-CVaR model generated the lowest Sharpe Ratio of the entire group (1.4333). This inefficiency is a direct result of its defensive behavior, where the model constantly shifts into concentrated corner solutions that increase standard volatility and miss broader market upside. The GRMVE framework decisively outperformed the Mean-CVaR model in mean-variance efficiency (Sharpe of 1.6105 vs 1.4333) while delivering slightly higher total returns.

Structural Superiority, Diversification and Turnover: The most compelling argument for the GRMVE framework emerges when analyzing the structural practicality of the portfolios. The Average HHI metric proves the immense value of integrating Yager’s entropy. The GRMVE model achieved an Average HHI of 0.0529. In a 20-asset universe, a perfectly equal-weighted portfolio has an HHI of 0.0500. This confirms that the GRMVE framework successfully forces a near-perfect structural diversification, actively preventing the concentration risk that standard Mean-Variance or models without diversification objective suffer from. By contrast, the Mean-CVaR model exhibited dangerous concentration, yielding an Average HHI of 0.1466 (nearly triple that of GRMVE).

Furthermore, this structural stability directly impacts trading costs. Because the asset universe dynamically changes every 6 months via the Calmar pre-selection stage, baseline turnover is required even for the Naive strategy (0.2500) just to transition to the new asset pool. The GRMVE framework navigated these transitions with a highly manageable Average Turnover of 0.3048. In glaring contrast, the Mean-CVaR model generated an extreme Average Turnover of 0.7727. This indicates that the Mean-CVaR model was aggressively liquidating and replacing over 75% of its portfolio weight during re-optimizing to chase optimal tail-risk scenarios, a hyper-activity that would incur devastating real-world transaction costs.

In summary, these metrics confirm that the GRMVE framework is a superior practical tool for institutional portfolio management. While the Naive strategy offers high raw returns but entirely ignores structural constraints and drawdown limits, and the Mean-CVaR model offers tail-risk protection at the cost of extreme concentration and prohibitive turnover, the GRMVE framework achieves the optimal balance. It delivers robust, highly efficient returns and downside protection, while explicitly guaranteeing a broadly diversified, low-turnover portfolio structure that can be reliably and cost-effectively implemented in dynamic real-world markets.

### 4.4. Statistical significance of performance differences

To rigorously validate the comparative performance of the GRMVE framework, we conducted three distinct statistical tests on the 18-month out-of-sample return series. Following the recommendations in the robust portfolio literature, we employed: (1) a paired t-test to evaluate differences in mean returns; (2) the Diebold-Mariano (DM) test to assess the predictive accuracy and differential of the return streams; and (3) the Jobson-Korkie (JK) test, specifically designed to test the statistical significance of differences in Sharpe Ratios.

The null hypothesis for all tests posits that there is no significant difference between the performance of the proposed GRMVE framework and the respective benchmark model. The results, including test statistics and p-values, are summarized in Table 4.

**Table 4:** Statistical Significance Tests (GRMVE vs. Benchmarks).

Benchmark	Paired t-test (p-value)	DM Test (p-value)	JK Test (p-value)
RMV	0.505 (0.620)	0.519 (0.603)	−0.025 (0.980)
MVE	0.095 (0.926)	0.097 (0.922)	−0.075 (0.940)
Mean-CVaR	−0.010 (0.992)	−0.010 (0.992)	0.208 (0.835)
Naive (1/n)	−1.336 (0.199)	−1.375 (0.169)	−0.702 (0.483)

*Note: The table reports the test statistic followed by the p-value in parentheses. All tests evaluate the 18-month out-of-sample period.

As shown in Table 4, all p-values across the three tests exceed the standard 0.05 (5% significance) threshold. Consequently, we cannot reject the null hypothesis; the out-of-sample returns and Sharpe Ratios of the GRMVE framework are not statistically distinguishable from the benchmark models over this 18-month evaluation horizon.

This lack of statistical significance is a well-documented phenomenon in portfolio optimization literature, most notably highlighted by DeMiguel et al. [6], who demonstrated the extreme difficulty of demonstrating statistically significant outperformance over the 1/n rule without excessively large sample sizes. Given the inherent volatility of financial data, an 18-month out-of-sample period possesses limited statistical power to detect marginal differences in mean-variance efficiency.

However, this statistical indistinguishability highlights the practical value of the proposed framework. The tests confirm that the GRMVE model successfully achieves the robust performance levels of the state-of-the-art Mean-CVaR model, and the famously resilient Naive strategy, without statistically underperforming them. Crucially, while the performance metrics (returns/Sharpe) are statistically tied, the GRMVE framework achieves these returns while satisfying strict, real-world practical constraints, such as manageable turnover and explicit diversification bounds, which the Naive strategy completely ignores, and which purely non-robust models (MVE) fail to stabilize. Therefore, the economic significance of the GRMVE model lies in its ability to match top-tier risk-adjusted performance while delivering a vastly superior, practically implementable portfolio structure.

### 4.5. Sensitivity analysis

To better understand the mathematical behavior and theoretical consistency of the proposed GRMVE framework, we conducted a rigorous sensitivity analysis. The purpose of this evaluation is to quantify how the model’s objective value and structural feasibility respond to simultaneous variations in its key constraints. Using the asset universe and historical estimates from the initial optimization window (Period 0), we evaluated 220 distinct parameter combinations across three critical dimensions:

1. *The trade-off controlling parameter* (λ): Ranging from 0.0 to 1.0 in increments of 0.1, this parameter balances the dual objectives of maximizing diversification via Yager’s entropy and minimizing portfolio variance.
2. *The budget of uncertainty* (Γ): Tested at discrete levels {1,3,5,7,10}, this integer sets the maximum number of assets allowed to simultaneously deviate to their worst-case returns, directly controlling the portfolio’s conservatism.
3. *The uncertainty magnitude parameter* (δ): Tested across four volatility scenarios {0.25,0.5,1.0,1.5}, this multiplier scales the maximum allowable deviation for each asset’s expected return ($d_{i}=\delta \sigma_{i}$), simulating increasingly hostile market shocks.

*Model Sensitivity to Risk-Diversification Preferences* (λ): When operating within a mathematically feasible space, the trade-off parameter λ serves as the primary driver of the objective function. As λ increases from 0 to 1, the model’s preference shifts from achieving a perfectly uniform distribution to seeking the absolute minimum variance portfolio. This inverse relationship confirms that λ acts as an effective, intuitive parameter for the investor, smoothly translating strategic risk preferences into precise structural allocations. However, as the following results demonstrate, the ability to act on this preference is strictly governed by the model’s robust constraints.

**Fig 6:**
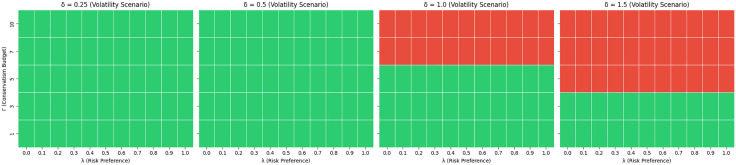
Feasibility Frontier Evolution.

The Feasibility Frontier and the Limits of Robustness: An important theoretical insight from this analysis is the identification of the model’s feasibility boundary. Out of the 220 parameter combinations tested, the framework successfully solved 165 feasible scenarios, while 55 scenarios were rendered mathematically infeasible. The visual evolution of this boundary is plotted in Fig 6 (Green indicates mathematically feasible portfolios while Red indicates infeasible combinations where robust constraints violate the target return).

Fig 6 reveals a clear hierarchical relationship that dictates portfolio viability. Feasibility is primarily constrained by the robustness parameters (Γand δ), which define the absolute size of the feasible solution space. As assumed market volatility (δ) increases from 0.25 to 1.5, the green feasible region systematically collapses.

The model becomes definitively infeasible under combinations of high conservatism (e.g., Γ≥7) and extreme market uncertainty (δ≥1.0). This highlights a fundamental mathematical trade-off inherent to robust optimization: high values of Γand δ enforce a massive penalty on expected returns to guarantee worst-case performance. The optimization solver fails exactly at the threshold where the robust return drops below the minimum required target return ($\mu_{\text{0}}$).

Interestingly, the investor’s risk preference (λ) also interacts with this boundary. A high λ pushes the model toward low-variance assets, which historically offer lower nominal returns. Therefore, portfolios with a high λ are often the first to become infeasible when the robust penalty is applied, as their baseline returns are too low to absorb the worst-case shock while still meeting the $\mu_{\text{0}}$ target.

#### 4.5.1. Summary and practical implications.

This sensitivity analysis reveals a critical hierarchy for portfolio managers. The level of conservatism (Γ) and expected market volatility (δ) establish a hard mathematical limit on what is achievable, while the risk preference (λ) guides the selection of the optimal portfolio within that limit.

These findings have profound practical implications for dynamic portfolio management. A practitioner seeking a highly defensive, low-variance portfolio (a high λ) must be willing to accept a more moderate budget of uncertainty (a lower Γ) during highly volatile market regimes to ensure a solution remains achievable. Conversely, if maximum worst-case protection is mandated, the manager must lower their target return expectations.

In the end, the GRMVE framework’s value lies not only in generating an optimal allocation, but in explicitly quantifying these trade-offs. This allows institutional investors to set realistic, mathematically sound strategic goals, and perfectly motivates the implementation of dynamic calibration techniques, such as adjusting Γ based on real-time volatility indices, to independently navigate this feasibility frontier in live markets.

## 5. Discussion

This study set out to address two critical gaps in the portfolio optimization literature: the need for an adaptive, multi-period robust framework and the integration of diversification measures with robust optimization approach. By extensively testing the proposed Generalized Robust Mean-Variance-Entropy (GRMVE) framework on a dynamically pre-selected universe of 60 S&P 500 stocks over a 72-month period, the numerical results definitively demonstrate that the GRMVE model provides a powerful and practical solution, achieving highly stable and compelling risk-adjusted performance in a realistic investment setting.

### 5.1. Interpretation of principal findings

The core finding of this research is that the simultaneous integration of parameter uncertainty and structural diversification goals within a multi-period framework leads to portfolios that are structurally superior to those omitting either element.

The value of our integrated approach is best understood by deconstructing its performance relative to the benchmarks. First, our comparison against the non-robust MVE model provided a compelling demonstration of the “error maximization” flaw. Even within an adaptive structure, the MVE model created unstable, concentrated portfolios by over-relying on potentially noisy historical data. The GRMVE framework, by accounting for input parameter uncertainty, completely avoided this weakness, resulting in superior risk-adjusted performance and exceptional downside protection.

Second, the integration of Yager’s entropy proved essential for structural practicality. While the purely robust RMV model successfully minimized downside risk, it did so without regard for concentration. The GRMVE framework’s entropy constraint actively forced a broadly diversified allocation (achieving an HHI of 0.0529, nearly identical to a perfectly equal-weighted portfolio).

Finally, our comparison against the state-of-the-art Mean-CVaR model yielded critical insights into the practical limitations of tail-risk models. While Mean-CVaR successfully mitigated the severe March 2025 market crash, it suffered from severe concentration and the highest trading turnover of any model. This frequent, extreme re-allocation caused the model to miss broader market recoveries. The GRMVE framework effectively occupied a highly efficient allocation strategy, matching the tail-risk protection of CVaR and the compounding growth of the broader market, while doing so with less than half the trading turnover.

Notably, while rigorous statistical testing (e.g., Jobson-Korkie and Diebold-Mariano tests) indicated that the raw risk-adjusted returns were not statistically distinguishable from the SOTA Mean-CVaR or Naive models over the 18-month horizon (p>0.05), this finding is highly significant from a practical standpoint. It confirms that the GRMVE framework successfully matches the return of the famously unbeatable Naive model and SOTA tail-risk models, but strictly enforces real-world constraints (cardinality, diversification bounds, controlled turnover) that purely heuristic or scenario-based models cannot reliably guarantee.

### 5.2. Practical and theoretical implications

The findings of this paper have significant implications for both financial practitioners and academic researchers.

For practitioners, the GRMVE framework offers a computationally tractable Mixed-Integer Quadratic Program (MIQP) that avoids the “black-box” interpretability issues of Machine Learning (ML) strategies and the extreme computational intractability of Dynamic Stochastic Programming (DSP). It provides a transparent, flexible tool for real-world portfolio management. The explicit trade-off identified between structural diversification (entropy) and trading costs provides managers with a quantitative framework for balancing portfolio stability against transaction costs.

Furthermore, while our empirical analysis utilized fixed parameters to establish a controlled baseline, the model’s structure natively supports dynamic parameter calibration. In live market settings, practitioners can employ adaptive schemes to continuously tune the framework. For example, the budget of uncertainty (Γ) could be dynamically scaled using external macroeconomic indicators, such as increasing Γ in proportion to the VIX (Volatility Index) to automatically enforce conservatism during market panics. Alternatively, rolling k-fold cross-validation could be utilized at each re-optimization step to select parameters that minimize out-of-sample validation error over the trailing window.

The primary theoretical contribution of this study is the development of an integrated framework that treats parameter uncertainty and concentration risk not as separate sequential problems, but as deeply interdependent dimensions of portfolio risk. This challenges the prevailing modular approach in the literature and demonstrates that simultaneous optimization leads to structurally more resilient allocation strategies.

### 5.3. Limitations of the study

While the empirical results are compelling, we acknowledge several limitations that contextualize our findings. First, although we expanded our dataset to include 60 assets spanning all 11 GICS sectors over a 72-month period, the analysis remains constrained to US large-cap equities. The findings may not be fully generalizable to alternative asset classes, such as international equities, fixed-income securities, commodities, or cryptocurrencies, which exhibit highly distinct correlation structures and non-normal return distributions.

Second, our transaction cost model assumed a linear, proportional fee structure. In institutional practice, transaction costs often involve non-linear market impact dynamics and bid-ask spread fluctuations during extreme market stress, which could further penalize high-turnover models like Mean-CVaR. Finally, the performance of the proposed framework was evaluated using static risk preferences (λ) across the out-of-sample period, whereas actual investor utility functions may shift dynamically with changing wealth levels and market regimes.

### 5.4. Future research directions

The limitations of this study suggest several rich opportunities for future research. A direct next step would be to test the GRMVE framework’s performance across diverse, multi-asset class portfolios. Additionally, future research should formally explore the integration of Machine Learning techniques for dynamic parameter calibration. Reinforcement Learning (RL) agents or regime-switching Hidden Markov Models (HMM) could be trained to identify market regimes and dynamically shift the risk-diversification trade-off (λ) and robustness budget (Γ) month-to-month, effectively transitioning the GRMVE framework into a fully autonomous, regime-aware asset management system. Finally, a comparative study integrating other advanced entropy measures, such as Rényi or Tsallis entropy, within the same multi-period robust MIQP framework could yield valuable insights into which mathematical definition of diversification provides the most effective out-of-sample stability.

## 6. Conclusion

This research was motivated by a persistent and critical gap in the portfolio optimization literature: the fact that methods for handling parameter uncertainty and ensuring structural diversification have largely evolved in parallel. We aimed to answer whether a unified, dynamic framework could overcome the individual limitations of existing models. To this end, we introduced and empirically validated the Generalized Robust Mean-Variance-Entropy (GRMVE) framework, a novel Mixed-Integer Quadratic Programming (MIQP) approach that simultaneously integrates budgeted uncertainty with an explicit Yager’s entropy diversification objective within an adaptive, multi-period setting.

Through a rigorous empirical analysis utilizing an expanding-window re-optimization approach over a 72-month horizon, the study provides strong evidence for the value of this integrated methodology. Our proposed GRMVE framework demonstrated a compelling ability to navigate severe market downturns and volatile recoveries. It successfully avoided the classic “error maximization” flaw that produced highly unstable portfolios in the non-robust MVE model. Furthermore, it proved structurally superior to the state-of-the-art Mean-CVaR model; while CVaR successfully insulated against tail-risk, it suffered from severe concentration and prohibitive trading turnover. The GRMVE framework effectively resolved these issues, achieving risk-adjusted returns mathematically comparable to the famously resilient Naive (1/n) and Mean-CVaR benchmarks, but doing so via a vastly more stable, highly diversified, and cost-effective allocation path that respects strict real-world constraints.

The primary theoretical contribution of this work is the demonstration that portfolio risk, arising from both parameter uncertainty and asset concentration, must be treated as an interdependent optimization problem. For practitioners, this research provides a computationally tractable, flexible tool that bridges the gap between sophisticated robust mathematics and realistic institutional implementation. Furthermore, the extensive sensitivity analysis offers a crucial insight into a mathematical “feasibility hierarchy.” We demonstrate that the level of conservatism (Γ) and market volatility (δ) establish a hard frontier on achievable out-of-sample outcomes, while the investor’s risk preference (λ) guides the selection of the optimal portfolio strictly within those bounds. This provides portfolio managers with a clear, quantitative framework for setting realistic strategic goals.

While the results of this expanded 72-month study are compelling, we acknowledge directions for future research. Although the asset universe was dynamically pre-selected across all 11 GICS sectors, the dataset was limited to US large-cap equities. A natural next step is to test the GRMVE framework’s performance across multi-asset class portfolios, including international markets, fixed income, and alternative assets with non-normal return distributions. Finally, the framework establishes a perfect foundation for the integration of Machine Learning. Future research should explore adaptive schemes, such as Reinforcement Learning or regime-switching models, to dynamically calibrate the model’s key parameters (λ and Γ) in response to real-time market volatility. By building on this work, the field can move towards more integrated, computationally tractable, and truly autonomous solutions for the long-standing challenge of dynamic portfolio optimization.

## Supporting information

S1 Appendix (DOCX)

## References

[1] Markowitz H. Portfolio Selection. J Finance. 1952;7(1):77–91. doi: 10.2307/2975974

[2] Ghanbari H, Mohammadi E, Fooeik AML, Kumar RR, Stauvermann PJ, Shabani M. Cryptocurrency Portfolio Allocation under Credibilistic CVaR Criterion and Practical Constraints. Risks. 2024;12(10):163. doi: 10.3390/risks12100163

[3] Kumar RR, Ghanbari H, Stauvermann PJ. Can Including Cryptocurrencies with Stocks in Portfolios Enhance Returns in Small Economies? An Analysis of Fiji’s Stock Market. JRFM. 2025;18(9):484. doi: 10.3390/jrfm18090484

[4] Best MJ, Grauer RR. On the sensitivity of mean-variance-efficient portfolios to changes in asset means: some analytical and computational results. Rev Financ Stud. 1991;4(2):315–42.

[5] Chopra VK, Ziemba WT. The Effect of Errors in Means, Variances, and Covariances on Optimal Portfolio Choice. JPM. 1993;19(2):6–11. doi: 10.3905/jpm.1993.409440

[6] DeMiguel V, Garlappi L, Uppal R. Optimal Versus Naive Diversification: How Inefficient is the 1/NPortfolio Strategy?. Rev Financ Stud. 2007;22(5):1915–53. doi: 10.1093/rfs/hhm075

[7] Ben-Tal A, Nemirovski A. Robust Convex Optimization. Math Oper Res. 1998;23(4):769–805. doi: 10.1287/moor.23.4.769

[8] Bertsimas D, Sim M. The price of robustness. Oper Res. 2004;52(1):35–53.

[9] Soyster AL. Convex programming with set-inclusive constraints and applications to inexact linear programming. Oper Res. 1973;21(5):1154–7.

[10] Philippatos GC, Wilson CJ. Entropy, market risk, and the selection of efficient portfolios. Applied Economics. 1972;4(3):209–20. doi: 10.1080/00036847200000017

[11] Bera AK, Park SY. Optimal Portfolio Diversification Using the Maximum Entropy Principle. Econometric Reviews. 2008;27(4–6):484–512. doi: 10.1080/07474930801960394

[12] Campbell JY, Viceira LM. Strategic asset allocation: portfolio choice for long-term investors. Clarendon Lectures in Economic. 2002.

[13] Yager R. Measures of entropy and fuzziness related to aggregation operators. Information Sciences. 1995;82(3–4):147–66. doi: 10.1016/0020-0255(94)00030-f

[14] Mossin J. Optimal Multiperiod Portfolio Policies. J BUS. 1968;41(2):215. doi: 10.1086/295078

[15] Merton RC. Lifetime portfolio selection under uncertainty: The continuous-time case. Rev Econ Stat. 1969;:247–57.

[16] Samuelson PA. Lifetime Portfolio Selection By Dynamic Stochastic Programming. The Review of Economics and Statistics. 1969;51(3):239. doi: 10.2307/1926559

[17] Pogue GA. An extension of the markowitz portfolio selection model to include variable transactions’ costs, short sales, leverage policies and taxes. The Journal of Finance. 1970;25(5):1005–27. doi: 10.1111/j.1540-6261.1970.tb00865.x

[18] Elton EJ, Gruber MJ. On the Optimality of Some Multiperiod Portfolio Selection Criteria. J BUS. 1974;47(2):231. doi: 10.1086/295633

[19] Aliaga-Diaz R, Renzi-Ricci G, Daga A, Ahluwalia H. Portfolio Optimization with Active, Passive, and Factors: Removing the Ad Hoc Step. JPM. 2020;46(4):39–51. doi: 10.3905/jpm.2020.1.127

[20] The Vanguard Asset Allocation Model: An investment solution for active-passive-factor portfolios. https://corporate.vanguard.com/content/dam/corp/research/pdf/the_vanguard_asset_allocation_model_an_investment_solution_for_active_passive_factor_portfolios.pdf. Accessed 2025 August 14.

[21] Woodside-Oriakhi M, Lucas C, Beasley JE. Portfolio rebalancing with an investment horizon and transaction costs. Omega. 2013;41(2):406–20. doi: 10.1016/j.omega.2012.03.003

[22] Wong Y-S, Hsieh C-H. On frequency-based log-optimal portfolio with transaction costs. IEEE Control Syst Lett. 2023;7:3489–94.

[23] Plaxco LM, Arnott RD. Rebalancing a Global Policy Benchmark. JPM. 2002;28(2):9–22. doi: 10.3905/jpm.2002.319828

[24] Dantzig GB, Infanger G. Multi-stage stochastic linear programs for portfolio optimization. Ann Oper Res. 1993;45(1):59–76. doi: 10.1007/bf02282041

[25] Brodt AI. A dynamic balance sheet management model for a Canadian chartered bank. Journal of Banking & Finance. 1978;2(3):221–41. doi: 10.1016/0378-4266(78)90013-4

[26] Glomb L, Liers F, Rösel F. A rolling-horizon approach for multi-period optimization. Eur J Oper Res. 2022;300(1):189–206.

[27] Lübbecke ME, Desrosiers J. Selected topics in column generation. Operations Research. 2005;53(6):1007–23.

[28] Ben-Tal A, Nemirovski A, El Ghaoui L. Robust optimization. 2009. https://www.torrossa.com/gs/resourceProxy?an=5576051&publisher=FZO137

[29] Calafiore GC. Multi-period portfolio optimization with linear control policies. Automatica. 2008;44(10):2463–73. doi: 10.1016/j.automatica.2008.02.007

[30] Ghaoui LE, Oks M, Oustry F. Worst-case value-at-risk and robust portfolio optimization: A conic programming approach. Operations Research. 2003;51(4):543–56.

[31] Bertsimas D, Shtern S, Sturt B. A Data-Driven Approach to Multistage Stochastic Linear Optimization. Management Science. 2023;69(1):51–74. doi: 10.1287/mnsc.2022.4352

[32] Ling A, Sun J, Wang M. Robust multi-period portfolio selection based on downside risk with asymmetrically distributed uncertainty set. Eur J Oper Res. 2020;285(1):81–95.

[33] Ma Y, Han R, Wang W. Portfolio optimization with return prediction using deep learning and machine learning. Expert Systems with Applications. 2021;165:113973. doi: 10.1016/j.eswa.2020.113973

[34] Black F, Litterman R. Global Portfolio Optimization. Financial Analysts Journal. 1992;48(5):28–43. doi: 10.2469/faj.v48.n5.28

[35] Kallberg JG, Ziemba WT. Mis-specifications in portfolio selection problems. In: Risk and Capital: Proceedings of the 2nd Summer Workshop on Risk and Capital Held at the University of Ulm, West Germany June 20–24, 1983, 1984. 74–87.

[36] Yam SCP, Yang H, Yuen FL. Optimal asset allocation: Risk and information uncertainty. Eur J Oper Res. 2016;251(2):554–61.

[37] Ben-Tal A, Nemirovski A. Robust solutions of linear programming problems contaminated with uncertain data. Math Program. 2000;88(3):411–24.

[38] Bertsimas D, Brown DB, Caramanis C. Theory and Applications of Robust Optimization. SIAM Rev. 2011;53(3):464–501. doi: 10.1137/080734510

[39] Larni-Fooeik A, Sadjadi SJ, Mohammadi E. Stochastic portfolio optimization: A regret-based approach on volatility risk measures: An empirical evidence from The New York stock market. PLoS One. 2024;19(4):e0299699. doi: 10.1371/journal.pone.0299699 38648229 PMC11034657

[40] Fabozzi FJ, Kolm PN, Pachamanova DA, Focardi SM. Robust portfolio optimization and management. John Wiley & Sons. 2007.

[41] El Ghaoui L, Oustry F, Lebret H. Robust Solutions to Uncertain Semidefinite Programs. SIAM J Optim. 1998;9(1):33–52. doi: 10.1137/s1052623496305717

[42] Goldfarb D, Iyengar G. Robust portfolio selection problems. Math Oper Res. 2003;28(1):1–38.

[43] Robust and data-driven optimization: Modern decision making under uncertainty. Models, methods, and applications for innovative decision making. INFORMS. 2006:95–122. doi: 10.1287/educ.1063.0022

[44] Gregory C, Darby-Dowman K, Mitra G. Robust optimization and portfolio selection: The cost of robustness. European Journal of Operational Research. 2011;212(2):417–28. doi: 10.1016/j.ejor.2011.02.015

[45] Sadjadi SJ, Gharakhani M, Safari E. Robust optimization framework for cardinality constrained portfolio problem. Applied Soft Computing. 2012;12(1):91–9. doi: 10.1016/j.asoc.2011.09.006

[46] Bertsimas D, Pachamanova D. Robust multiperiod portfolio management in the presence of transaction costs. Comput Oper Res. 2008;35(1):3–17.

[47] Chiou WJP, Lee AC, Chang CCA. Do investors still benefit from international diversification with investment constraints? Q Rev Econ Finance. 2009;49(2):448–83.

[48] Kapur JN. Maximum-entropy models in science and engineering. John Wiley & Sons. 1989.

[49] Shannon CE. A mathematical theory of communication. Bell Syst Tech J. 1948;27(3):379–423.

[50] Simonelli MR. Indeterminacy in portfolio selection. Eur J Oper Res. 2005;163(1):170–6.

[51] Zheng Y, Zhou M, Li G. Information entropy based fuzzy optimization model of electricity purchasing portfolio. In: 2009 IEEE Power & Energy Society General Meeting, 2009. 1–6. https://ieeexplore.ieee.org/abstract/document/5275643/

[52] Usta I, Kantar YM. Mean-Variance-Skewness-Entropy Measures: A Multi-Objective Approach for Portfolio Selection. Entropy. 2011;13(1):117–33. doi: 10.3390/e13010117

[53] Lassance N, Vrins F. Portfolio selection: A target-distribution approach. European Journal of Operational Research. 2023;310(1):302–14. doi: 10.1016/j.ejor.2023.02.014

[54] Mercurio PJ, Wu Y, Xie H. An Entropy-Based Approach to Portfolio Optimization. Entropy (Basel). 2020;22(3):332. doi: 10.3390/e22030332 33286106 PMC7516790

[55] Rényi A. On measures of entropy and information. In: Proceedings of the fourth Berkeley symposium on mathematical statistics and probability, 1961. 547–62.

[56] Lassance N, Vrins F. Minimum Rényi entropy portfolios. Annals of Operations Research. 2021;299(1–2):23–46. doi: 10.1007/s10479-019-03364-2

[57] Pola G. On entropy and portfolio diversification. J Asset Manag. 2016;17(4):218–28. doi: 10.1057/jam.2016.10

[58] Tsallis C. Possible generalization of Boltzmann-Gibbs statistics. J Stat Phys. 1988;52(1–2):479–87. doi: 10.1007/bf01016429

[59] Ribeiro M, et al. The entropy universe. Entropy. 2021;23(2):222. 33670121 10.3390/e23020222 PMC7916845

[60] Devi S. Financial portfolios based on Tsallis relative entropy as the risk measure. J Stat Mech. 2019;2019(9):093207. doi: 10.1088/1742-5468/ab3bc5

[61] Gaied Chortane S, Naoui K. The end of mean-variance? Tsallis entropy revolutionises portfolio optimisation in cryptocurrencies. J Risk Financ Manag. 2025;18(2):77.

[62] Wu J, Sun B-L, Liang C-Y, Yang S-L. A linear programming model for determining ordered weighted averaging operator weights with maximal Yager’s entropy. Computers & Industrial Engineering. 2009;57(3):742–7. doi: 10.1016/j.cie.2009.02.001

[63] Yu J-R, Lee W-Y, Chiou W-JP. Diversified portfolios with different entropy measures. Appl Math Comput. 2014;241:47–63.

[64] Rockafellar RT, Uryasev S. Optimization of conditional value-at-risk. JOR. 2000;2(3):21–41. doi: 10.21314/jor.2000.038

[65] Sharpe WF. Mutual Fund Performance. J Bus. 1966;39(1):119–38.

[66] Sortino FA, Price LN. Performance Measurement in a Downside Risk Framework. JOI. 1994;3(3):59–64. doi: 10.3905/joi.3.3.59

