# Supplementary material for: A multi-period robust portfolio optimization framework using yager’s entropy
Source: PLoS One. 2026 May 14;21(5):e0332725. doi: 10.1371/journal.pone.0332725 (PMC13175339; doi:10.1371/journal.pone.0332725)
Supplement: S1 Appendix — (DOCX) [file pone.0332725.s001.docx]

Appendix

Step 1: The Classical Mean-Variance Model

In order to build the proposed model of this paper, we use a well-known single objective variant of the standard Markowitz mean-variance model which seeks to find an optimal portfolio allocation that minimizes portfolio risk (variance) for a predetermined level of expected return. The nominal formulation of this problem is a quadratic program:

| *Minimize:* | $\sum_{i=1}^{n} \sum_{j=1}^{n} x_{i}x_{j}{Cov}_{i,j}$ | (34) |
| --- | --- | --- |
| *s.t.* |  |  |
|  | $\sum_{i=1}^{n} \mu_{i}x_{i}\geq\mu_{0}$ | (35) |
|  | $\sum_{i=1}^{n} x_{i}=1$ | (36) |
|  | $x_{i}\geq0 ; \forall i\in\{1,\ldots,n\}$ | (37) |

Where $x_{i}$ is the portfolio weight allocated to asset $i$, $\mu_{i}$ is the nominal expected return of asset $i$, and $\mu_{0}$ is the minimum required portfolio expected return. The primary limitation of this model is its reliance on point estimates for expected returns, which are subject to significant uncertainty in practice [6].

Step 2: Defining the Uncertainty Model for Returns

To account for uncertainty, we assume that each expected return $\tilde{\mu_{i}}$, is an uncertain parameter that belongs to a symmetric, bounded interval; $\tilde{\mu_{i}}=\left[ \mu_{i}-d_{i}, \mu_{i}+d_{i} \right]$ where $d_{i}$ represents the maximum possible deviation from the nominal return, quantifying the magnitude of uncertainty for asset $i$.

Step 3: Formulating the Robust Counterpart with a Budget of Uncertainty

The core of the robust methodology is to ensure that the portfolio constraints are satisfied even under adverse, but controlled, scenarios. The Bertsimas and Sim [8] framework introduces a budget of uncertainty, denoted by $\Gamma$, a parameter that controls the level of conservatism. The parameter Γ constrains the number of asset returns that can simultaneously deviate from their nominal values to have the worst possible impact on the constraint. The robust counterpart of the expected return constraint requires that the constraint holds true for all possible realizations of returns within the defined uncertainty set. This leads to the following worst-case formulation of the return constraint:

|  | $\sum_{i=1}^{n} \tilde{\mu_{i}}x_{i}\geq\mu_{0}$ | (38) |
| --- | --- | --- |

Given the uncertainty definition and the budget Γ, the worst-case (minimum) portfolio return is realized when up to Γ returns deviate to their lowest possible value ($\mu_{i}-d_{i}$). The expression for the worst-case portfolio return becomes:

|  | $\sum_{i=1}^{n} \mu_{i}x_{i}-\{max\sum_{i=1}^{n} d_{i}x_{i}\}\geq\mu_{0}$ | (39) |
| --- | --- | --- |

The term max $\{max\sum_{i=1}^{n} d_{i}x_{i}\}$ represents the maximum potential loss from the nominal return, given that at most $\Gamma$ assets can deviate.

Step 4: Derivation of the Tractable Linear Counterpart

The formulation in Step 3 is not a standard linear constraint due to the max operator. To create a tractable model, we leverage strong duality theory to convert this expression into a set of linear constraints. The maximization term is the optimal value of the following linear program:

| *Maximize:* | $\sum_{i=1}^{n} (d_{i}x_{i})y_{i}$ | (40) |
| --- | --- | --- |
| *s.t.* |  |  |
|  | $\sum_{i=1}^{n} y_{i}\leq\Gamma$ | (41) |
|  | ${0\leq y}_{i}\leq1$ | (42) |

The dual of this problem provides an equivalent minimization formulation. By strong duality, we can replace the maximization term with its dual, introducing new auxiliary variables $z$ and $p$. The dual problem is:

| *Minimize:* | $z\Gamma+\sum_{i=1}^{n} p_{i}$ | (43) |
| --- | --- | --- |
| *s.t.* |  |  |
|  | $z+p_{i}\geq d_{i}x_{i} ; \forall i$ | (44) |
|  | $z, p_{i}\geq0 ; \forall i$ | (45) |

It must be noted that since the non-negativity constraint $x_{i}\geq0$ is active, $\left| x_{i} \right|$ simplifies to $x_{i}$.

*Theory*: From Primal to Dual

The process of formulating the dual problem involves a systematic transformation of the primal problem’s components. Essentially, the roles of the objective function coefficients and the right-hand side constants of the constraints are interchanged. Also, the constraints of the primal become variables in the dual, and the variables in the primal become constraints in the dual.

| Primal Problem (Maximization):   \| *Maximize:* \| $Z=c^{T}x$ \| \| --- \| --- \| \| s.t. \|  \| \|  \| $Ax\leq b$ \| \|  \| $x\geq0$ \|   Where:   - $x$ is the vector of decision variables ($n\times1$). - $c$ is the vector of coefficients in the objective function ($n\times1$). - $A$ is the matrix of coefficients in the constraints ($m\times n$). - $b$ is the vector of right-hand side values of the constraints ($m\times1$). - $m$ is the number of constraints and $n$ is the number of variables. | Dual Problem (Minimization):   \| *Minimize:* \| $W=b^{T}y$ \| \| --- \| --- \| \| s.t. \|  \| \|  \| $A^{T}y\geq c$ \| \|  \| $y\geq0$ \|   Where:   - $y$ is the vector of dual variables ($m\times1$). - $A^{T}$is the transpose of the matrix $A$. |
| --- | --- | --- | --- | --- | --- | --- | --- | --- | --- | --- | --- | --- | --- | --- | --- | --- | --- |

Step 5: The Final Robust Mean-Variance Formulation

By substituting the linearized dual formulation back into the portfolio optimization problem, we obtain the final Robust Mean-Variance model. This model is a standard quadratic program and can be solved efficiently.

| *Minimize:* | $\sum_{i=1}^{n} \sum_{j=1}^{n} x_{i}x_{j}{Cov}_{i,j}$ | (46) |
| --- | --- | --- |
| *s.t.* |  |  |
|  | $\sum_{i=1}^{n} \mu_{i}x_{i}-(z\Gamma+\sum_{i=1}^{n} p_{i})\geq\mu_{0}$ | (47) |
|  | $\sum_{i=1}^{n} x_{i}=1$ | (48) |
|  | $z+p_{i}\geq d_{i}x_{i} ; \forall i\in\{1,\ldots,n\}$ | (49) |
|  | $x_{i},z, p_{i}\geq0 ; \forall i\in\{1,\ldots,n\}$ | (50) |

This final formulation selects a portfolio that is robust against estimation errors in expected returns.
